# Metabolomic Profiling of Bipolar Disorder by ^1^H-NMR in Serbian Patients

**DOI:** 10.3390/metabo13050607

**Published:** 2023-04-28

**Authors:** Katarina Simić, Zoran Miladinović, Nina Todorović, Snežana Trifunović, Nataša Avramović, Aleksandra Gavrilović, Silvana Jovanović, Dejan Gođevac, Ljubodrag Vujisić, Vele Tešević, Ljubica Tasic, Boris Mandić

**Affiliations:** 1 Institute of Chemistry, Technology and Metallurgy, National Institute, University of Belgrade, Studentski trg 12-16, 11000 Belgrade, Serbia; katarina.simic@ihtm.bg.ac.rs (K.S.); ninat@chem.bg.ac.rs (N.T.); dgodjev@chem.bg.ac.rs (D.G.); 2 Institute of General and Physical Chemistry, Studentski trg 12-16, 11158 Belgrade, Serbia; zmiladinovic@iofh.bg.ac.rs; 3 University of Belgrade - Faculty of Chemistry, Studentski trg 12-16, 11000 Belgrade, Serbia; snezanat@chem.bg.ac.rs (S.T.);; 4 University of Belgrade - Faculty of Medicine, Institute of Medical Chemistry, Višegradska 26, 11000 Belgrade, Serbia; 5 Special Hospital for Psychiatric Diseases “Kovin”, Cara Lazara 253, 26220 Kovin, Serbia; gavrilovicaleksandra74@gmail.com (A.G.); silvana.jovanovic555@gmail.com (S.J.); 6 Institute of Chemistry, Organic Chemistry Department, State University of Campinas, Campinas 13083-970, SP, Brazil

**Keywords:** bipolar disorder, metabolomics, biomarkers, NMR, chemometrics, serum metabolites

## Abstract

Bipolar disorder (BD) is a brain disorder that causes changes in a person’s mood, energy, and ability to function. It has a prevalence of 60 million people worldwide, and it is among the top 20 diseases with the highest global burden. The complexity of this disease, including diverse genetic, environmental, and biochemical factors, and diagnoses based on the subjective recognition of symptoms without any clinical test of biomarker identification create significant difficulties in understanding and diagnosing BD. A ^1^H-NMR-based metabolomic study applying chemometrics of serum samples of Serbian patients with BD (33) and healthy controls (39) was explored, providing the identification of 22 metabolites for this disease. A biomarker set including threonine, aspartate, gamma-aminobutyric acid, 2-hydroxybutyric acid, serine, and mannose was established for the first time in BD serum samples by an NMR-based metabolomics study. Six identified metabolites (3-hydroxybutyric acid, arginine, lysine, tyrosine, phenylalanine, and glycerol) are in agreement with the previously determined NMR-based sets of serum biomarkers in Brazilian and/or Chinese patient samples. The same established metabolites (lactate, alanine, valine, leucine, isoleucine, glutamine, glutamate, glucose, and choline) in three different ethnic and geographic origins (Serbia, Brazil, and China) might have a crucial role in the realization of a universal set of NMR biomarkers for BD.

## 1. Introduction

Bipolar disorder (BD) is a mental disease that includes episodes of mania, depression, and euthymia, and it affects 1–3% of the population worldwide [1,2,3,4]. Bipolar disorder commonly runs in families: 80 to 90 percent of individuals with bipolar disorder have a relative with bipolar disorder or depression. Environmental factors such as stress, sleep disruption, and drugs and alcohol may trigger mood episodes in vulnerable people. Though the specific causes of bipolar disorder within the brain are unclear, an imbalance in brain chemicals is believed to lead to dysregulated brain activity. The average age of onset is 25 years old. Due to the involvement of diverse genetic, environmental, and biochemical factors, BD is a heterogenous illness for which diagnosis exclusively relies on the subjective recognition of symptoms without any objective methods such as the identification of biomarkers. Accordingly, inadequate treatments and deficient clinical outcomes are very often seen in patients with BD [5]. Early and precise diagnosis of BD is pivotal to improving the clinical treatment of BD patients [6]. In order to overcome that barrier, the identification of objective biomarkers obviously has a crucial and challenging role.

Metabolomics studies the alteration of small molecule metabolites in cells, tissues, and body fluids and explores the biochemical pathways related to the pathogenesis of disease while determining the objective biomarkers [7]. In addition to genomics, proteomics, and lipidomics, metabolomics is a valuable research method assessed by nuclear magnetic resonance spectroscopy (NMR), gas chromatography coupled to mass spectrometry (GC-MS), and liquid chromatography coupled to mass spectrometry (LC-MS) [8,9,10,11,12]. NMR spectroscopy is the main tool to explore metabolites due to its many advantages, such as the simple preparation of samples, high reproducibility supporting extensive metabolome analysis, and the possibility to analyze in vivo and ex vivo samples, which is crucial for clinical research [9,10,11,12,13,14,15,16,17]. The state-of-the-art advantages of NMR-based metabolomics is reflected in the possibility of its application in precision medicine through personalized medical treatment, reliable monitoring of treatment response, and clinical outcomes. Blood and urine samples can be obtained at minimal risk and cost; they are easily accessible and ideal for the identification and determination of new biomarkers [18]. In the last decade, a panel of potential biomarkers was explored and identified in biological fluids (blood, urine, cerebrospinal fluid) of BD patients applying ^1^H-NMR-based metabolomics [19,20,21,22,23,24,25,26,27,28,29,30]. Reported metabolomics studies demonstrated that diverse BD metabolites are connected with their altered biochemical pathways, including mitochondrial/energy metabolism, oxidative stress, amino acid metabolism, and lipid metabolism.

In this work, we studied the ^1^H-NMR-based metabolomics of human blood serum of BD patients in Serbia in order to identify alterations of metabolites. Our goal was to confirm the difference between BD patients and healthy control groups based on their metabolic profiles with the intention of identifying potential biomarkers for BD diagnosis and verifying the possibility of their use in personalized medicine. Additionally, our aim was to establish a better comprehension of biochemical pathways affected by BD.

## 2. Materials and Methods

### 2.1. Sampling and Sample Preparation

This study was approved by the Ethics Committee of the Special Hospital for Psychiatric Diseases “Kovin”, the University of Belgrade—Faculty of Chemistry, and the Blood Transfusion Institute of Serbia. Blood samples of selected BD patients under medical treatment were obtained from the Special Hospital for Psychiatric Diseases “Kovin”, while samples of healthy controls were provided by the Blood Transfusion Institute. All participants or their caretakers provided written consent prior to their enrollment in this study. Thirty-three BD patients, including 14 males and 19 females, with ages between 20 and 74 years, were analyzed in this research. The control group comprised 39 healthy volunteers, males (27) and females (12), with ages between 23 to 60 years old. The blood samples were collected in triplicates. They were kept on ice for one hour, centrifuged, and then the sera collected from supernatants were stored at −80 °C. The maximum period of storage before assays was up to two weeks. Serum samples were thawed and diluted with D_2_O (vol., 1:1) prior to NMR analyses.

### 2.2. NMR Analysis

All NMR experiments were carried out on a Bruker Avance ⅡI NMR spectrometer (Bruker BioSpin, Rheinstetten, Germany) operating at 500.26 MHz for ^1^H, using BBI probe head and at temperature of 298 K. A one-dimensional 1H-NMR NOESY spectrum with presaturation during relaxation delay was acquired by standard noesypr1d pulse sequence for each sample. All 1D NOESY spectra were measured with the following acquisition parameters: power level for presaturation of 39.67 dB, 32 K complex data points, 128 scans, and a bandwidth of 15 ppm. The receiver gain was determined automatically before each measurement. Prior to Fourier transformation, the FIDs were weighed by an exponential function with the line-broadening factor of 0.3 Hz. The methyl of lactate at 1.33 ppm (3H, 3J = 7.0 Hz) was used as a referent chemical shift signal. Additionally, 2D experiments TOCSY, HSQC, and JRES were used for metabolites’ assignment. The TOCSY experiments were measured by mlevphpr.2 pulse sequence with 2048 complex data points, 512 increments, 32 scans, and mixing time of 160 ms. The HSQC experiment was recorded applying hsqcetgpprsisp2.2 pulse sequence, with 1024 complex data points, 256 increments, and 120 scans. JRES spectra were measured using jresgpprqf pulse sequence, 16 K complex data points, 40 increments, and 128 scans. All applied pulse sequences were taken from the Bruker library. Together with the 2D experiment assignments and interpretation, the literature and available databases, such as HMDB (Human Metabolome Database), were used to assist in the assignment of molecules.

### 2.3. Chemometrics

#### 2.3.1. Software

Data processing in this work was entirely carried out using toolboxes and software implementations, including custom scripts and codes created and run under MATLAB version 9.7 (MathWorks Inc., Natick, MA, USA) [31]. General NMR Analysis Toolbox (GNAT) version 1.2 [32] was used to read and reprocess ^1^H NMR, Noesy1d FID files into a MATLAB workspace. Alignment of all ^1^H NMR spectra before any further treatment was implemented through Interval Correlation Optimized Shifting (icoshift) version 3.0 beta [33]. Data pretreatment and further chemometrics analysis were accomplished by PLS Toolbox version 8.9.1 (Eigenvector Research, Inc., Manson, WA, USA) [34].

#### 2.3.2. Reading in Data

A series of recorded Bruker data files was first imported into GNAT Matlab software package [32], which performed some basic processing steps. The final size of each transformed spectrum was 32 K real data points. Phase correction was also carefully conducted before data exporting into the Matlab workspace. The dataset object is assembled as a total number of samples for both classes given in rows and 32,768 of the total number of observed variables expressed as NMR chemical shifts in columns of the dataset table. Furthermore, the same dataset object also includes accompanying categorical and class variables (sample labels, axis scale, response class variable, triplicate grouping variable, cross-validation index variable, etc.) necessary for further chemometric analysis [35]. As a result, the data set table comprises 116 samples organized in triplicates from 39 patients belonging to the ‘Control’ healthy group and 102 samples of triplicates from 34 patients of the ‘BD’ group of patients, constituting 218 samples overall.

For unsupervised PCA analysis, a complete data set containing all samples in the data table was utilized. On the other hand, in the case of supervised OPLS-DA analysis, the initial data set (after the removal of identified outlier samples) was divided into two parts: the training (calibration) part, which accounts for approximately 2/3 of the complete number of samples in the data table, keeping the same ratio of the number of class samples as an initial data set (stratify option); and the test data set used for external validation of OPLS-DA models that incorporates the remaining (approximately 1/3 of complete) samples of the initial data set. As a result of the splitting procedure, the training data set consists of 143 total samples: 77 samples from 26 individuals of ‘Control’ group and 66 samples from 22 patients of the ‘BD’ group, whereas the test data set includes 71 samples among which 33 samples from 11 patients of the ‘BD’ group and 38 samples from 13 ‘Control’ group individuals. All samples in both data sets are grouped as triplicates for each certain patient, and their allocation inside the data set was assigned to a specific categorical variable [35].

#### 2.3.3. Peak Alignment

Prerequisite that any chemometric methods of analysis could be applicable to the NMR spectral data set is so called bilinearity, meaning that each column (if samples are stored in the rows of a matrix) contains information originating from the same resonant signal along all the samples in data table [33]. Commonly, misalignment of ^1^H NMR spectra was overcome using bucketing or binning techniques for this purpose. However, bucketing performs a data reduction by grouping spectral responses, since it is not strictly a method to align data [36]. In addition, observed loadings obtained from fully aligned spectra and data-reduced spectra clearly demonstrate the benefit of using as high a spectral resolution as possible since reduced spectra can lead to imprecise or incomplete interpretation [37]. To fulfill this precondition, the alignment of ^1^H NMR spectra in this work icoshift [33] alignment procedure was exploited. The median spectrum from the current data set was chosen as a reference target vector. The shift of the whole spectra according to a reference signal in the region 5.20 to 5.35 ppm was performed using 4 iterations. All other details regarding the alignment procedure using icoshift in this work can be found in our previous publication [35].

#### 2.3.4. Data Pretreatment (Preprocessing)

Before further data treatment, the region between 4.35 and 5.0 ppm (water suppression signals residuals) was removed from the data set. A first-order polynomial baseline function was used to baseline the spectra, which were then fitted to regions without peaks and then subtracted from the original spectra. Normalization was carried out using probabilistic quotient normalization (PQN) [38]. Results in our previous work also indicate that spectral regions below 0.17 ppm and above 8 ppm should be excluded [35]. In this way, the variation originating from these omitted areas was significantly reduced. Overall, the data matrix of an initial 32 K was reduced to 15,180 data points in the second dimension.

#### 2.3.5. Centering and Scaling

Prior to any modeling, the centering and scaling of data should be performed. Since the results of any method of centering and scaling depend on the number of samples to which relate, any centering and scaling were incorporated during model assembling and subsequent cross-validation.

For centering, the mean centering of each column variable in the data set table that was used provides a mean vector with the same length as the axis scale of ^1^H NMR spectrum. This kind of centering was commonly used prior to Pareto scaling (scaling to the square root of each variable standard deviation) or scaling to the standard deviation of each column variable of the data set table (so-called auto-scale centering and scaling). In addition, so-called class centroid centering, which centers data to the centroid of all classes, was also used for further comparison and analysis in conjunction with pooled standard deviation [34,35,39].

Class centroid centering and scaling are useful for centering and scaling in cases where the sample subset represents different population subsets, and the subsets are very unbalanced. Using the class centroid avoids the mean being dominated by the most populous subset [34]. Pooled standard deviation is a weighted average of the standard deviation (variances) from the same population subsets. Therefore, the larger subset size causes a proportionally greater effect/impact on the overall estimate of standard deviation and consequently scaling results.

Different kinds of scaling, as was shown [37,40,41], have a significant impact on the real shape of resulting model loadings and consequently could lead to misinterpretation of the true significance of some spectral variables. Autoscaling (mean-centering and scaling to standard deviation of all samples’ variables in the data set) gives the same weight to all the spectral variables because of their now equal variance, and therefore, the resulting loadings indicate only the variables, which really impact the discrimination between classes [37]. On the other hand, Wiklund et al. [41] proposed using Pareto scaling rather than UV scaling, suggesting a positive impact on the models’ predictive ability as a consequence of a reduction in the noise and artifacts in the models. Therefore, due to the strong distortion caused by the variance scaling procedure in the case of autoscaling, Pareto scaling could provide some advantages over autoscaling regarding the interpretation of the obtained loadings. Nevertheless, it still keeps the limitation of distorted loadings, and the high variance variables have relatively more weight in the modeling [37]. In such a situation, the problem of more or less distorted loading plots as a consequence of different scaling methods could be overcome with back-scale projection methods proposed by Cloarec et al. [37,40], which will be used for further loading interpretation in the present work as well [35].

#### 2.3.6. Cross-Validation (CV)

In this study, 7-fold continuous block cross-validation (CV) was used according to the method adopted and developed in our previous study [35]. The data set is split into the CV blocks with randomly reordered samples (shuffling) inside each of the blocks preserving the triplicate structures of samples.

## 3. Results

A total of 33 patients with BD and 39 healthy control participants were included in this study. Blood samples from both groups were prepared in triplicate for NMR analyses. The summary of the collected sample characteristics is presented in Table 1.

### 3.1. Chemometrics

The ^1^H-NMR data sets were transported into a matrix, and chemometrics analyses were performed using the GNAT Matlab software package. Spectra phases and baselines were corrected using automatic options, while 0th-order phase correction was carried out manually in order to remove the contribution of noise.

#### 3.1.1. Exploratory Analysis

In order to determine the presence of potential outliers or to find optimal methods for scaling variables in the data set, performing an exploratory analysis was always recommended [42]. However, simple univariate methods are not easily applicable when the data set has a large number of variables, as in the case of NMR data sets. Nevertheless, univariate statistics such as skewness and kurtosis, or comparative analysis of standard deviations, were found to be informative to some extent and helpful in determining the method of scaling or variable regions with significant discrepancy from normal distribution [35].

Figure S1a (Supplementary Information) depicts a comparison of standard deviations between both classes in the data set. In addition, Figure S1b shows a comparison between standard deviations that takes into account all samples in the dataset and the pooled standard deviation determined according to the following expression:

(1) $$s_{pool}=\sqrt{\frac{\left(n_{1}-1\right)\cdot s_{1}^{2}+\left(n_{2}-1\right)\cdot s_{2}^{2}+\cdots +\left(n_{m}-1\right)\cdot s_{m}^{2}}{n_{1}+n_{2}+\cdots +n_{m}-m}}$$

whereas *s*_1_, *s*_2_, … *s_m_* represent standard deviations of corresponding variables related to a particular class; *n*_1_, *n*_2_, … *n_m_* represent the number of samples in each of classes and *m* is the total number of classes.

As can be seen from Figure S1b, the most pronounced difference (region specified with arrows) in standard deviations and the pooled standard deviation could be observed in the range of 3.62 to 3.73 ppm. This result suggests that the method of scaling could be of great importance for the understanding of obtained results during further multivariate modeling. Therefore, for the purpose of this study, we have utilized Pareto scaling, scaling to the unit variance, and scaling to the pooled variation (pooled standard deviation).

#### 3.1.2. PCA Models

Principal Component Analysis (PCA) is also a well-known unsupervised chemometric analytical technique that is useful for exploratory data analysis. As a result of their application, a reduced number of orthogonal principal components representing linear combinations (weighted average of the original variables) of original variables are obtained [43,44]. Projection of the predictor data matrix on such reduced hyperspace provides PCA scores values assembled into the matrix with the number of samples given as rows and the number of latent variables given as columns.

In order to detect reliable outliers, establishing the number of components for each of the PCA models was necessary [44]. The number of components in each of the PCA models was determined based on the Scree test and the minimum for Root Mean Squared Error of Cross-Validation (RMSECV). As a result, all PCA models presented in this work were composed using six principal components. The PCA model, wherein the data prior to modeling was mean-centered and Pareto-scaled, captured 91.82% of the total variance. The separation between classes occurred along the PC 2 component (explained 11.31% of the total variance), while the first two components explained 73.28% of the total variance after removing outlier samples. Variance captured by the first two components was given in Figure S2, accompanied by PC 2 back-scale projected loadings.

PCA model accomplished using class centroid centering and scaling, where all other preprocessing parameters were the same as for the model with Pareto scaling and mean centering, was explained 83.93% of total variance by six components. Very distinct class separation, as in the case of Pareto scaling, was now observed along the PC1 component (captured 34.94 % of total variance). The results of PCA analysis for this kind of scaling and centering are presented in Figure S3.

The principal components analysis (PCA) of the ^1^H-NMR spectra data set, in combination with the plot of Q residuals against T^2^ Hotelling (influence plot [44]), allowed us to identify potential outliers. Figure S4a shows the influence plot obtained during the assembling PCA model preprocessed by class centroid centering and scaling. Clearly, three scores of BD samples belonging to the same patient (all triplicates) could be identified, along with a-single sample from the ‘Control’ group. All four samples have distinctive separation, either regarding Q residual or T^2^ Hotelling values in comparison to other samples, for all assembled PCA models regardless of the method of centering and scaling. A closer examination of the T^2^ contribution plot for samples of the ‘BD’ group indicates several chemical shifts area (centered around 0.89, 1.29, 1.58, 2.04, 2.24, 2.75, 4.07, 4.27, 5,23 and 5.23 ppm) where these particular samples show in corresponding ^1^H NMR spectra much higher intensity values in comparison to the other samples. Therefore, these outliers were removed from the data set for the rest of the study.

In addition, the score plot of the resulting PCA model preprocessed using class centroid and centering (depicted in Figure S4b), after excluding identified outliers, indicates that variability within the same group of samples triplicates (belonging to the same patient), is on average much smaller than the variability between the groups of samples of patient. Therefore, to perform data set splitting for the test and training groups of samples for the purpose of CV, a triplicate of the same patient should be kept together inside each test or calibration group of samples, which was implemented throughout this work.

**Figure 1:**
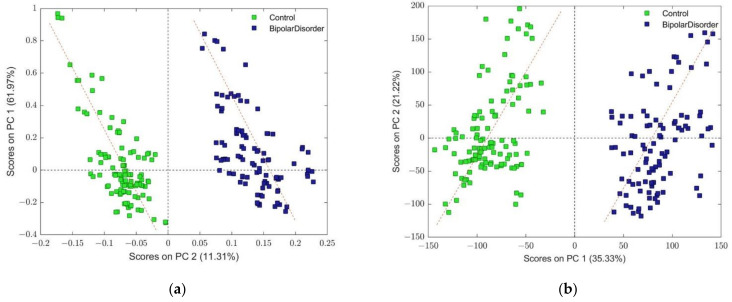
(**a**) Score plot of PCA model composed with mean centering and Pareto scaling, where dashed line denotes the direction of cluster shapes for each of the classes, which is tilted slightly left in comparison to the vertical PC1 component. (**b**) Score plot of PCA model composed with class centroid and centering, where dashed line denotes the direction of cluster shapes for each of the classes, which is tilted slightly right in comparison to the vertical PC2 component. Both models were assembled after removing identified outliers.

It can be seen from Figures S2a and S3a a good separation between the two main classes. However, corresponding component loadings for these two models (Figures S2b and S3b) show a slight discrepancy in the related observed variable’s contribution to the given principal component (PC). The reason for such behavior could be found in the observed tilt angle regarding global scores swarm orientation relative to vertical PC components for both models (but in opposite directions (see Figure 1)). In order to interpret the component that is considered relevant, the first step is generally followed by a rotation of the components that were retained in the model [45,46]. Since the rotations are always performed in a subspace, the new axes will always explain less variance than the original components (which are computed to be optimal), but obviously, the part of variance explained by the total subspace if the rotation is the same as it was before rotation (only the partition of the variance between components has changed) [45]. Orthogonal rotation of loadings belonging to the models depicted in Figure 1 was performed according to the varimax algorithm (Kaiser-Varimax rotation [47]) as a part of the PLS toolbox.

The number of subspace components used as input for varimax rotation was at least the first two components (PC 1 and PC 2 in the case of the PCA model where the data set was Pareto-scaled) or all six components of the PCA model where the data set was preprocessed by class centroid centering and scaling. Score plots obtained after the projection of preprocessed original data onto factor space defined with new loadings were utilized in order to verify the reliability of the varimax rotation method. The results of rotation analysis for corresponding loadings are presented in Figure S5 (Supplementary Information). As can be seen from the figure, both loadings, although related to different model components (depending on the centering and scaling method), are almost identical regarding each variable contribution to the particular component. Therefore, the resulting loadings plot could be used for the more reliable assignation of groups of variables/chemical shifts (pertaining to metabolites molecules structure) that contribute to the class separation.

A closer inspection of the loadings from Figure S5 reveals that the most positive contribution, corresponding to the class ‘BD’, could be found at the position of 0.856 ppm and 1.246 ppm, as well as in the range between 3.76 and 3.60 ppm, which could be assigned to the resonances of sugar molecules. Doublet centering around 1.328 ppm (d: 1.321 ppm; 1.335 ppm), assigned to lactate, also has a significant impact. In addition, corresponding variables in PC loadings found at 3.216 ppm and 3.553 ppm show the characteristic dispersion-phase signature of the chemical shift variation [35]. The aforementioned variables/chemical shifts are very similar to the findings from our previous paper related to the discrimination of the ‘Schizophrenia’ group of patients (see Figure 1b from [35]). Nevertheless, a comparison of the corresponding loadings of these two groups of patients reveals that the area between 4.012 ppm and 4.146 ppm showed the most significant differences in their loading shape. Quartet peaks centered around 4.11 ppm (q: 4.089 ppm; 4.104 ppm; 4.117 ppm; 4.132 ppm), which also belong to lactate, show dispersion behavior (one side peaks at 4.089 ppm and 4.104 ppm have a positive sign, while the other peaks at 4.117 ppm and 4.132 ppm have a negative sign). Three new peaks that appear also show positive loading contributions. The first distinctive peak centered at 4.038 ppm, the additional two at 4.075 ppm, and another overlapped by a lactate quartet at 4.09 ppm. The correct assignment of these three latter peaks is challenging due to their proximity to or overlapped with the lactate quartet. The next noticeable difference between these two loadings is several very sharp negatively signed resonances positioned in the range of 3.324 to 3.546 ppm and 3.785 to 3.942 ppm.

#### 3.1.3. OPLS-DA Models

**Figure 2:**
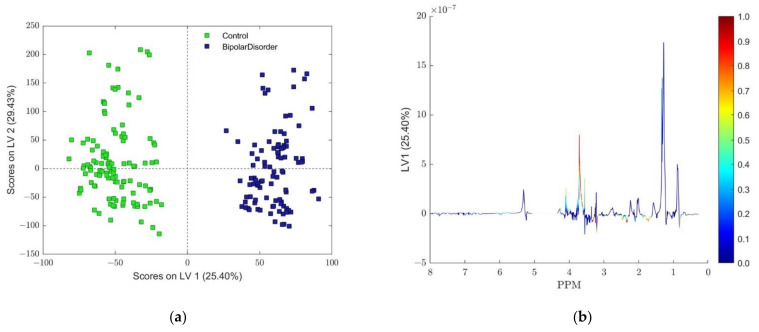
(**a**) Scores plot of the first two LV components of the OPLS-DA model using mean-centering and unit variance scaling. The BD samples are shown in dark blue, and the control group samples are shown in light green. (**b**) Back-scale projection of loading vector LV 1 to coloring coded according to the absolute value of particular loading weighted by correlation of spectral data set and scores matrix from the OPLS-DA model.

**Figure 3:**
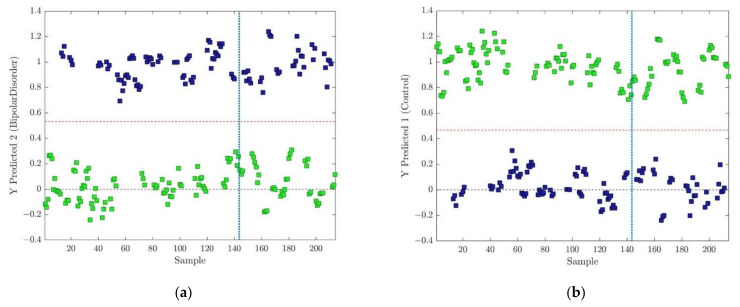
(**a**) Classification results for prediction of training (**left** from blue dot line) and test (**right** of the blue dotted line) data set for the ‘BD’ group of patients, and the threshold value of 0.5326. (**b**) Classification results for prediction of training (**left** from blue dot line) and test (**right** of the blue dotted line) data set for the ‘Control’ group of individuals and the threshold value of 0.4674. Auto centering and scaling were performed as data preprocessing.

In any metabonomic study, identifying the molecules that have significant importance on the metabolomic pathway, which is characteristic of the problem under investigation, is crucial. Thus, PLS-DA and, subsequently, OPLS-DA regression methods, among others, enable the discrimination of diverse classes of samples and, at the same time, the identification of statistically relevant compounds responsible for such differentiation. In that sense, understanding how much the loading amplitudes of related samples variable contribute to a particular model latent variable represents the primary goal of such investigation. However, the method of scaling and centering of the initial data set has a strong impact on the shape of the resulting loading vector. As a consequence, interpretation can be distorted because some metabolites with apparent covariation in the loadings are not really responsible for the discrimination between the different groups or classes [37]. As explained in the experimental section, different kinds of scaling and centering were tested during model composing. The OPLS-DA approach, as with all other regression methods, is sensitive to model complexity. To estimate the relevant number of components in all presented OPLS-DA models, as exemplified in Figure 2, a 7-fold CV was used. Prediction capabilities were tested for the chosen number of components with an independent test data set comprising 33 samples of ‘BD’ and 38 samples belonging to the ‘Control’ class, a total of 71 samples, as illustrated in Figure 3. The misclassification error, i.e., classification error rate (proportion of samples which were incorrectly classified) and the class error (average of false positive rate and false negative rate for class) [48] were used as primary metrics to compare model performance and the number of chosen components. As a result, the final number of components was selected as a compromise between misclassification and class error, and the minimum value of RMSECV was obtained for a different number of model components.

All OPLS-DA model using mean centering and unit variance scaling was accomplished using three latent variables (one predictable and two orthogonal). The score plot of the first predictive LV 1 component (comprising 25.40% of the variance) and the first orthogonal LV 2 component (comprising 29.43% of the variance) are depicted in Figure 2. The total captured variance by the OPLS-DA model was 61.74% by the X block and 94.50% by the Y block of the data set. Back scale projection of the predictive component was given with color coding according to the loading correlation vector proposed by Wiklund et al. [41], also known as an S line plot [49].

Predictions based on the CV for the training data set and predicted results for the test data set for both classes using autoscaling as a preprocessing method for centering and scaling are presented in Figure 3. The classification threshold for each class model was calculated using the Bayesian method [50]. For the ‘BD’ and ‘Control’ classes, the thresholds were determined as 0.5326 and 0.4674, respectively. The obtained accuracy of 1.0 for all models points to perfect class separation.

#### 3.1.4. Variable Importance Signature

To improve visualization of the variable influence in a model, several techniques were proposed that basically rely on a combination of the covariance and correlation loading profiles obtained from a projection-based model, e.g., the predictive component t_p_ of an OPLS-DA model [41]. Cloarec et al. [37] proposed a method for the examination of variable importance obtained from the OPLS-DA model, using the loadings from auto-scaled models plotted after back transformation with the respective weight of each variable. Since autoscaling, as a result, gives the same weight to all the spectral variables, distorted loadings and high variance variables have a relatively small influence during modeling. In this way, a loading plot (covariance) with the same shape as that of an NMR spectrum, presented on the same plot with important variables for the discrimination between the classes (correlation), highlighted by the color code, allows a direct interpretation of such loadings as pseudo-NMR spectra [37]. In a similar manner, combining the contribution or magnitude (covariance) with the effect and reliability (correlation) for the model variables, with respect to model component scores, provides an opportunity for a different method of predictive component loading visualization [41]. Hence, the covariance between score vector t_p_ and mean-centered data set X corresponds to the back-transformed loading vector of the OPLS-DA predictive component, when used in conjunction with the correlation between the same vectors, produced similar results to those of the Cloarec method. Figure 2b depicts the back-scale projection of loading vector LV 1 now using the absolute value of the correlation vector for color coding of loadings, also known as the S line plot [49].

#### 3.1.5. VIP Scores

**Figure 4:**
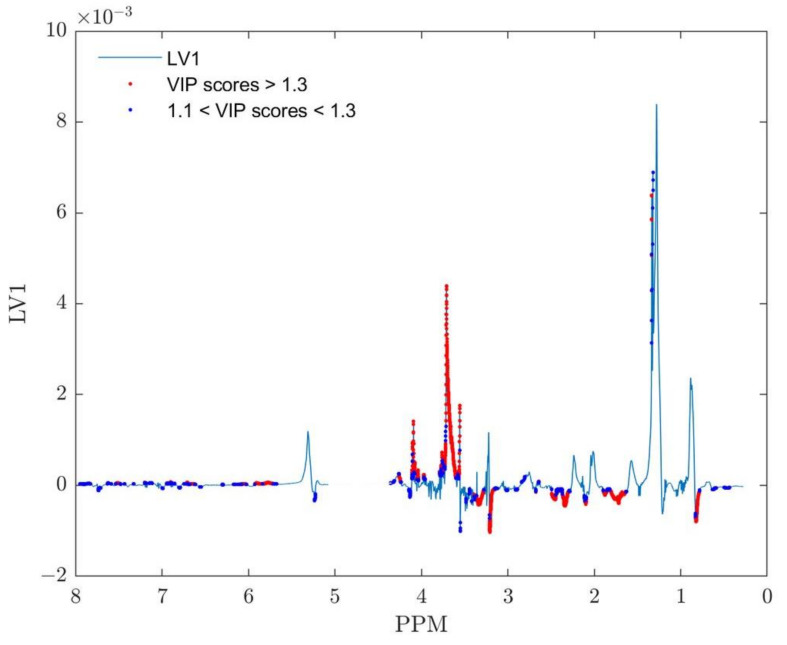
VIP scores presented on OPLS-DA model back-scale projection of LV1 predicting component using auto-scale centering and scaling for preprocessing. VIP scores > 1.3 are marked as red dots; blue dots represent VIP scores ranged between 1.1 to 1.3.

On the other hand, Variable Importance in Projection (VIP) [49], represents the most frequently used method for variable discrimination (variable discriminatory analysis) in chemometrics. VIP stands for a weighted combination over all components of the squared PLS weights, where the weighting that takes place is based on the explained sum of squares of response variable Y [51]. Since both PLS loadings and weights are strongly influenced by the method of scaling of the initial data set, a similar effect could be expected to be reflected in results for VIP scores too. For the same (covariance) loading obtained from the mean-centered X data matrix [41], the influence of two different centering and scaling methods on resulting VIP scores is presented in Figure 4.

The threshold value of 1.33 (from Figure 4) was determined by removing the VIP scores of the OPLS-DA model obtained after class centroid centering and scaling from the VIP scores values of the OPLS-DA model composed after the data set was auto-scaled. The maximum value of the remaining set of VIP scores of the auto-scaled model represents the obtained threshold. The resulting set of VIP scores also only includes values greater than 1.1 [52]. Variables assigned to the VIP scores in the range between 1.1 and 1.33 should be considered for further chemical validation. VIP scores greater than 1.33 were regarded as important for metabolite identification and class discrimination. In such a way, it was possible to examine the VIP scores for both of these models at the same time and gain insight into how the scaling method affected the outcomes of the variable importance (in projection) analysis.

Both methods (color-coded back-scale projection loadings and VIP scores) presented in Figure 2b and Figure 4 indicate almost identical ranges of chemical shifts inside NMR spectra as potential biomarker assignment areas for the distinction between two classes of samples: ‘BD’ and ‘Control’. The most pronounced difference could be recognized in the range 3.61–3.73 ppm belonging to sugar molecules, and according to the loadings plot, it is more relevant to class ‘BD’ than for the ‘Control’ class. Although the doublet assigned to lactate centered around 1.328 ppm shows smaller VIP scores and correlation loading values, their corresponding lactate quartet centered at 4.11 ppm deserves particular attention during chemical validation. The spectral parts between 1.63–1.80 ppm, 2.30–2.37 ppm, 2.45–2.50 ppm, 3.16–3.21 ppm, 3.28–3.35 ppm, and 3.36–3.38 ppm could be clearly identified from the back-scaled plot in Figure 2b and connected to the class ‘Control’. A nearly identical conclusion could be derived from the VIP score plot (Figure 4) for scores higher than 1.3, suggesting that both methods reveal nearly identical variables. It should be emphasized that findings obtained from the analysis of PCA re-scaled loadings (Figure S5) indicate almost the same final interpretation.

### 3.2. NMR Analyses

The results of chemometric analyses with VIP values higher than 1.3 were obtained using 1D Nuclear Overhauser enhancement spectroscopic data (NOESY1D). In accordance with these results, the metabolites as potential biomarkers in serum samples of the BD patients from a Serbian cohort were identified by performing analyses of spectral 2D NMR data, which was accomplished in TOCSY, JRES, and HSQC experiments. The set of 22 metabolites as serum BD biomarkers are presented with spectral data in Table 2.

## 4. Discussion

**Figure 5:**
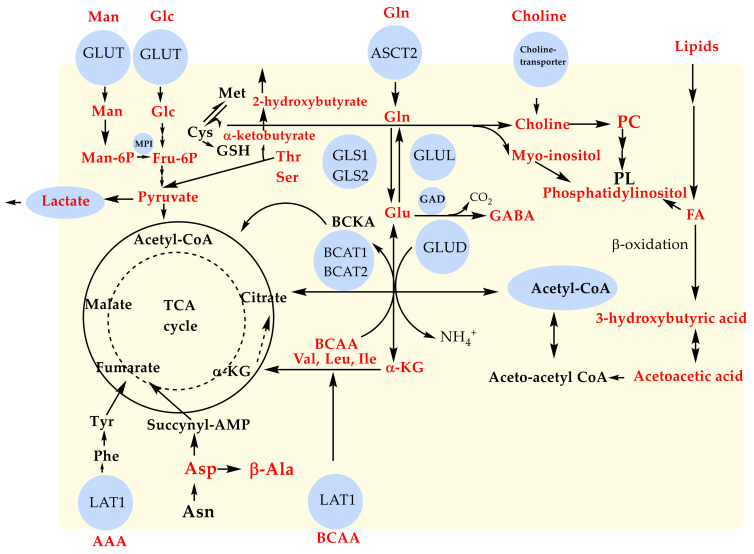
Illustration of the main metabolic pathways reported as altered in BD. Glucose (Glc), Mannose (Man), Glutamine (Gln), Glutamate (Glu), α-ketoglutarate (α-KG), γ-aminobutyric acid (GABA), Choline, Phosphatidylcholine (PC), Phospholipids (PL), Fatty acids (FA), Branched Chain Amino Acids (BCAA, such as valine—Val, leucine—Leu, isoleucine—Ile), Cysteine (Cys), Methionine (Met), Glutathione (GSH), Threonine (Thr), Serine (Ser), Asparagine (Asn), Aspartate (Asp), Aromatic Amino Acids (AAA, such as phenylalanine—Phe and tyrosine-Tyr), and many metabolic enzymes, such as glutaminase (GLS1 and GLS2), glutamine synthetase (GLUL), glutamate dehydrogenase (GLUD), branched chain aminotransferase (BCAT1 and BCAT2), glutamate decarboxylase (GAD), mannose-phosphate isomerase (MPI), and transporters, such as the transporter of glucose (GLUT), glutamine (ASCT2), and L-type amino acid transporter 1 (LAT1), are illustrated.

The NMR-based metabolomic profiling of serum from BD samples of Serbian patients and healthy controls provided the identification of 22 metabolites as biomarkers panel for this mental disease (Table 2 and Table 3). Threonine, aspartate, gamma-aminobutyric acid (GABA), 2-hydroxybutyric acid, serine, and mannose were confirmed for the first time in the BD serum samples by an NMR-based metabolomics study (Figure 5).

Changes in these biomarkers obviously confirmed the alteration of amino-acids metabolism, TCA cycle, and glycolysis. When the glucose metabolism is disturbed for supplying energy, other sources are used by tissues [24,25,26,27,28]. For the first time, our results showed an alteration of mannose, C-2 epimer of glucose, which also plays a role in energy generation. Mannose is mostly catabolized by mannose-6-phosphate isomerase (MPI) in fructose-6-phosphate, and it is then used in several metabolic pathways, including glycolysis (Figure 5) [53]. Mannose impacts the regulation of calcium signaling by alteration of neurotransmission and synaptic plasticity. Actually, Xu et al. reported that mannose induces depressive/anxiety-like behavior and spatial memory impairment in mice [53]. Reported data confirmed a high correlation between serine and threonine metabolism (Figure 5) and pyruvate metabolism with bipolar disorder [29]. Maes et al. showed significant alteration of serine and threonine in patients with treatment-resistant depression [54]. Yoshima et al. established a significant decrease in the serum level of serine in BD compared to those in healthy control participants [29]. On the other hand, the level of L-serine in patients with schizophrenia was increased [55,56], indicating the possibility of the implication of L-serine as a biomarker in psychiatric disorders. Transamination of branched-chain amino acids (BCAA) has an important role in the production of GABA in the brain (Figure 5). GABA (an established biomarker in our study) is known as a neurotransmitter affecting common excitatory processes regarding simple receptors that increase the flow of positive ions by opening ion channels [26]. Additionally, 2-hydroxybutyric acid is an identified biomarker in this study, obtained by the reduction of alpha-ketobutyrate, which is produced by amino acid catabolism (threonine and methionine) and glutathione anabolism (Figure 5) [35]. Furthermore, 2-hydroxybutyric acid is correlated with deficient energy metabolism and impaired glucose regulation, causing the rise of enhanced lipid oxidation and oxidative stress [57]. Moreover, aspartate, a metabolite established in this research, is involved in the Krebs cycle, indicating an alteration of the TCA cycle in BD [29]. Aspartate, which is catabolized in β-alanine and also across succinyl-AMP byproduct, is involved in the TCA cycle producing fumarate (Figure 5). Increased concentrations of urinary 2-hydroxybutyrate [23] and β-alanine [22] were also found in BD patients, as well as in MDD patients [21] compared to healthy controls, indicating that an increase of 2-hydroxybutyrate levels might be correlated to increased oxidative stress in BD patients.

Nine biomarkers (lactate, alanine, valine, leucine, isoleucine, glutamine, glutamate, glucose, and choline) were previously identified in serum samples of patients from Brazil and China [24,25,26,27], and they are common metabolites for all three origins. These established biomarkers pointed to altered glycolysis, lipid metabolism, amino acids metabolism, urea cycle, and TCA cycle (Figure 5). Chen et al. identified 36 metabolites that differ in urine samples of BD patients compared to HC using a combination of GC-MS and NMR [23]. Reported data pointed out 2,4,4-dihydroxypyrimidine, one of metabolite in glutamine anabolism, as a highly accurate BD urinary biomarker indicating alteration of glutamine metabolism [21,22,23]. In addition to 2,4-dihydroxypyrimidine, azelaic acid, β-alanine, and pseudouridine were identified as other urinary potential biomarkers in BD [22,23]. Tasic et al. [24,25,26] established a set of 33 biomarkers based on 1D and 2D NMR analyses (CPMG, HSQC, and HMBC) of a Brazilian cohort of BD patients’ serum samples (Table 3). Guo et al. [27] analyzed serum samples of BD patients with non-suicidal self-injury (NSSI) (n = 31) patients with BD without NSSI (n = 46) and healthy controls by 1D NMR CPMG experiments and obtained 33 serum biomarkers for BD patients from a Chinese cohort (Table 3). Eight differential biomarkers (HDL, 3-hydroxybutyric acid, pyruvic acid, oxidized glutathione, glycerol, citrulline, creatinine, and β-glucose) were found in the serum of BD patients with NSSI and healthy controls impacting two important metabolic pathways, the urea and glutamate metabolism cycles [27]. On the other hand, eight metabolites (HDL, pantothenate, alanine, *N*-acetyl-glycoproteins, glycerol, dimethylglycine, ascorbate, and valine) in serum samples were confirmed to distinguish BD patients without NSSI from healthy controls, including glycine and serine metabolism pathway, and the glucose-alanine cycle [27].

Altered levels of glucose and lactate as a product of glucose catabolism revealed disturbed energy metabolism in BD patients [24,25,26,27], showing an agreement with our findings. Lan et al. [20] have also obtained increased levels of lactate in post-mortem brain tissue in BD patients, while Yoshimi et al. [19] pointed to alteration in the citric acid cycle in serum and CSF in male BD patients. Acetoacetate is a precursor of 3-hydroxybutyric acid (a biomarker also found in our study) obtained by its reduction (Figure 5), reflecting the possibility that ketone bodies might turn into an energy source when there is a lack of sufficient amounts of glucose in BD [35]. All these studies confirmed the important role of disturbed energy metabolism in the diagnosis of BD patients, and energy insufficiency might be correlated to the most common depressive symptoms of bipolar disorder [28]. The lipid-metabolism-related molecule found in our work, as well as in Brazilian and Chinese studies [24,25,26,27], was choline (Figure 5). Choline is a main component of lipids of cell membranes, and it has an important role as a precursor of the neurotransmitter acetylcholine participating in cholinergic neurotransmission [58]. There is clinical evidence that lecithin as a choline precursor is reasonably efficient in some patients with mania. Also, myo-inositol is a sugar that affects the metabolism of phospholipids and phosphoinositide’s second messenger pathway [26].

Following our results, altered levels of amino acids (alanine, valine, leucine, isoleucine), glutamine, and glutamate were also found in Brazilian and Chinese groups [26,27] when sera of BD patient samples were compared to controls under the different treatments pointing out disturbance of amino acid metabolism (Figure 5). Pålsson et al. [59] reported that glutamate and glutamine are well-known biomarkers with increased levels in blood serum and cerebrospinal fluid (CSF) of BD patients using HPLC with fluorescence detection and a possible explanation of their enhancement was related to mitochondrial dysfunction [60]. The BCAAs (valine, leucine, and isoleucine), as well as aromatic amino acids (AAAs, phenylalanine, and tyrosine), are biomarkers established in this work. BCAAs are essential amino acids that have the same carrier for their transport into the brain as aromatic amino acids (AAAs, phenylalanine, tyrosine, and tryptophan) [61]. Therefore, the rivalry between BCAAs and AAAs might affect the synthesis of some neurotransmitters, particularly dopamine, norepinephrine, and 5-hydroxytryptamine (serotonin) [61]. Therefore, the increased levels of BCAAs in the blood can impact neurotransmitter levels in the brain that influence brain function. Moreover, BCAA transamination has an important role in the production of glutamate and gamma-aminobutyric acid (GABA) in the brain, as well as in ammonia detoxification to glutamine in astrocytes (Figure 5).

## 5. Conclusions

^1^H-NMR metabolomic profiling in a cohort of Serbian patients with BD pointed out 22 biomarkers for bipolar disorder. Six of these biomarkers (threonine, aspartate, GABA, 2-hydroxybutyric acid, serine, and mannose) were established for the first time in serum samples of BD patients applying NMR analyses accomplished with chemometrics. NMR-based metabolomics study of BD patients from Serbia identified nine metabolites: lactate, alanine, valine, leucine, isoleucine, glutamine, glutamate, glucose, and choline; these results are the same as previously reported studies in serum samples of BD patients in Brazil and China, emphasizing their crucial role in the possibility of application as biomarkers for diagnosis of BD, reliable monitoring of treatment response, and clinical outcomes. The essential requirement to achieve the universality of the serum biomarkers for BD is to explore a unified analysis of data of different geographical and ethnic origins, taking into account larger sample sizes and the effects of medical treatments on BD patients.

## Figures and Tables

**Table 1:** Demographic characteristics of the sample.

	Patients	Control Group
Number of samples	33	39
Age in years	20–74	23–60
Sex (male/female)	14/19	27/12
BMI (Body mass index)	18.5–35.5	22.2–33.2
Smoker/non-smoker	22/11	19/20

**Table 2:** Metabolites/biomarkers identified in serum samples of the BD patients with spectral data.

No	Metabolites/Biomarkers	TOCSY Correlations (δ_H_, ppm)	JRES ((δ_H_ (ppm), Multiplicity, J (Hz))	HSQC (δ_H_/δ_c_ (ppm))
1	Lactate/lactic acid	4.10; 1.31	CH_3_: 1.31, d, 6.98; CH: 4.10 q, 7.0	1.32/22.79, 4.098/71.25
2	Threonine	1.31; 3.56; 4.24	CH_3_: 1.32, d, overlapped with lactate; CH: 3.56 d, 5.0; CH_2_: 4.23 dd, 4.9, 6.6, overlapped with acylglycerol	1.34/22.54, 3.55/63.42, 4.24
3	Leucine	0.95; 1.71; 3.71	CH_3_: 0.94, d, 6.24; CH_3_: 0.95, d, 6.24	0.94/23.41, 0.95/24.72, 1.71/42.70, 3.71
4	Valine	0.98; 1.03; 2.27; 3.62	CH_3_: 0.97, d, 7.00; CH_3_: 1.03, d, 7.00; CH: 3.59 d, 4.39	0.97/19.26, 1.02/20.6, 2.27, 3.59/63.27
5	Glutamine	2.12; 2.44; 3.74	CH_2_: 2.12 m; CH_2_: 2.44 m	2.12/29.27, 2.43/33.61, 3.74/57.11
6	Glutamate/glutamic acid	2.05; 2.35; 3.75	CH_2_: 2.04, m and 2.11 m	2.0/29.68, 2.34/36.28, 3.74/57.11
7	Citrate/citric acid	2.51; 2.68	CH_2_: 2.51 d, 16.0; CH_2_: 2.68 d, 16.0	-
8	Aspartate/aspartic acid	2.68; 2.80; 3.88	CH_2_: 2.66, dd, 8.8, 17.5 and 2.80, dd 3.8, 17.4	3.80/54.56
9	Alanine	1.46; 3.77	CH_3_: 1.46, d, 7.26	3.76/53.21
10	3-Hydroxybutyric acid	1.19; 2.34; 4.12	CH_3_: 1.19 d, 6.4; CH_2_: 2.40, dd, 7.2, 14.4 and 2.29 dd, 6.4, 14.4	-
11	Gamma-aminobutyric acid	1.9; 3.03	CH_2_: 3.04, t, 7.6	-
12	Choline	3.50; 4.05	CH_2_: 4.05 m	4.05/58.35
13	Glucose (α + β)	3.40; 3.52; 3.7; 3.75; 5.10; 5.22	CH-4: 3.40 m; CH-2: 3.52 dd, 3.7, 9.7; CH-3: 3.70 m (overlapped); CH_2_-6: 3.75 dd, 5.1, 12.0 and 3.83 m; CH-5: 3.82 m; CH-1: 5.22 d, 3.9	-
14	Arginine	4.07; 4.27; 5.20	3.23 t, 6.6; 1.70, m and 1.64, m	-
15	Lysine	1.70; 1.89; 3.03; 3.74	1.91 m	-
16	2-Hydroxybutyric acid	-	CH_3_: 0.88, t, 7.50; CH_2_: 1.70, m and 1.64, m or arginine	-
17	Isoleucine	-	CH_3_: 0.92, t, 7.4; CH_3_: 0.99, d, 7.0; 3.65 d, 4.04	-
18	Serin	-	CH_2_: 3.97, dd, 3.8, 12.2 and 3.92, dd 5.7, 12.2; CH: 3.82 overlapped	3.95/62.94, 3.81/59.2
19	Mannose	-	CH: 3.55 t, 9.4; CH: 3.79 m; CH: 3.84 dd, 2.2, 4.0; CH: 3.95 m; CH: 5.17, d1.4	-
20	Glycerol	-	CH_2_: 3.64 and 3.55 m; CH: 3.70 m (overlapped)	3.63 and 3.55/65.31
21	Tyrosine	6.88; 7.18	CH: 3.96, dd, 5.0, 8.1 or phenylalanine; Ar: 6.88 and 7.18	3.95/58.78, Ar: 6.88/118.6, 7.18/133.4
22	Phenylalanine	7.30; 7.36; 7.42	Ar: 7.30 m, 7.37 m, 7.41 m	Ar: 7.31/132.01, 7.40/131.80

**Table 3:** Metabolites/biomarkers identified in serum samples of Serbian, Brazilian, and Chinese patients with BD by NMR analyses.

No	Metabolites/Biomarkers	Serbian Serum Samples	Brazilian Serum Samples	Chines Serum Samples	References
1	Lactate/lactic acid	+	+	+	[24,25,26,27]
2	Threonine	+	−	−	-
3	Leucine	+	+	+	[24,25,27]
4	Valine	+	+	+	[24,25,26,27]
5	Glutamine	+	+	+	[24,25,26,27]
6	Glutamate/glutamic acid	+	+	+	[24,25,26,27]
7	Citrate/citric acid	+	−	+	[27]
8	Aspartate/aspartic acid	+	−	−	-
9	Asparagine	−	+	−	[26]
10	Alanine	+	+	+	[24,25,26,27]
11	3-Hydroxybutyric acid	+	−	+	[27]
12	Gamma-aminobutyric acid	+	−	−	-
13	Choline	+	+	+	[24,26,27]
14	Glucose	+	+	+	[24,27]
15	Arginine	+	+	−	[26]
16	Lysine	+	+	−	[26]
17	2-Hydroxybutyric acid	+	−	−	-
18	Isoleucine	+	+	+	[25,27]
19	Serin	+	−	−	-
20	Mannose	+	−	−	-
21	Glycine	−	+	−	[25]
22	Glycerol	+	−	+	[27]
23	Tyrosine	+	+	−	[25]
24	Phenylalanine	+	+	−	[25]
25	N-Acetyl-aspartyl-glutamic acid	−	+	−	[24,25]
26	N-Acetyl-phenylalanine	−	+	−	[24]
27	Ethanol	−	+	−	[25]
28	α-ketoglutaric acid	−	+	−	[24]
29	Lipoamide	−	+	−	[24,26]
30	Myo-inositol	−	+	+	[24,25,26,27]
31	Lipids	−	+	−	[24,25,26]
32	Proline	−	+	−	[24,26]
33	Glycoprotein lipids	−	+	−	[26]
34	Acetate	−	+	+	[26,27]
35	α-ketoisovaleric acid	−	+	−	[24]
36	Acetoacetate	−	−	+	[27]
37	Methionine	−	−	+	[27]
38	Guanidinoacetate	−	−	+	[27]
39	Uracil	−	−	+	[27]
40	Histidine	−	+	+	[25,27]
41	Taurine	−	−	+	[27]
42	Betaine	−	−	+	[27]
43	Acetone	−	−	+	[27]
44	2,3-diphospho-D-glyceric acid	−	+	−	[25]
45	monoethyl malonate	−	+	−	[25]
46	6-hydroxydopamine	−	+	−	[25]
47	Acetyl-choline	−	+	+	[25,27]
48	Fatty acids	−	+	−	[25]
49	Creatine	−	+	+	[24,25,27]
50	N-acetyl glycoproteins	−	−	+	[27]
51	O-acetyl glycoproteins	−	−	+	[27]
52	Pantothenate	−	−	+	[27]
53	Dimethylglycine	−	−	+	[27]
54	Citrulline	−	−	+	[27]
55	Ascorbate	−	−	+	[27]
56	HDL	−	−	+	[27]
56	Pyruvic acid	−	−	+	[27]
58	Oxidized GSH	−	−	+	[27]

## Data Availability

Data available on request due to restrictions, e.g., privacy or ethical. The data presented in this study are available on request from the corresponding author. The data are not publicly available due to privacy and ethical restrictions.

## Supplementary Materials

The following supporting information can be downloaded at: https://www.mdpi.com/article/10.3390/metabo13050607/s1, Figure S1: (a) Standard deviation along all variables (chemical shifts) in the data set for the BD samples group. (b) Standard deviation and pooled standard deviation for all variables in the data set considering all samples from both groups (BD and controls) depending on calculation methods. Arrows in inset plots indicate the ppm range where differences between these two classes were observed in the data set; Figure S2: (a) PCA score plot of the model obtained using mean centering and Pareto scaling. Samples rounded by dotted red ellipses from the BD patient group were also identified on the influence plot as potential outliers. A Hoteling T2 Confidence limit of 95% was also presented in the plot with the blue dashed line. (b) Loading plot of PC 2 component using mean centering and Pareto scaling; Figure S3. (a) PCA score plot of the model obtained using class centroid centering and scaling. Samples marked with a pink color rounded by dotted red ellipses from the BD group were also identified on the influence plot as potential outliers. (b) Loading plot of PC1 component using class centroid centering and scaling; Figure S4: (a) Influence plot for PCA model with class centroid centering and scaling. Three samples from the class ‘BD’ show large Hotelling T^2^ reduced values compared to other samples, and one from the class ‘Control’ shows higher values for both statistics. The reduced Q and T^2^ are normalized statistics divided by the confidence limit calculated from each model’s particular data and parameters. (b) PCA score plot of the model obtained using class centroid centering and scaling after removal of outlier samples. Scores of samples belonging to the different patients are grouped together and denoted with corresponding symbols and colors connected by lines among samples of each patient; Figure S5: Loading plot of PC1 component from model preprocessed by class centroid and centering and PC2 component from model preprocessed by mean centering and Pareto scaling varimax rotation of initial PCA model loadings. Resulting loadings are back-transformed (multiplying all values by their respective standard deviation).
