# Supplementary material for: Metabolomic Profiling of Bipolar Disorder by 1H-NMR in Serbian Patients
Source: Metabolites. 2023 Apr 28;13(5):607. doi: 10.3390/metabo13050607 (PMC10223505; doi:10.3390/metabo13050607)
Supplement: Supplementary file 1 [file metabolites-13-00607-s001.zip › metabolites-2348723-supplementary.pdf]

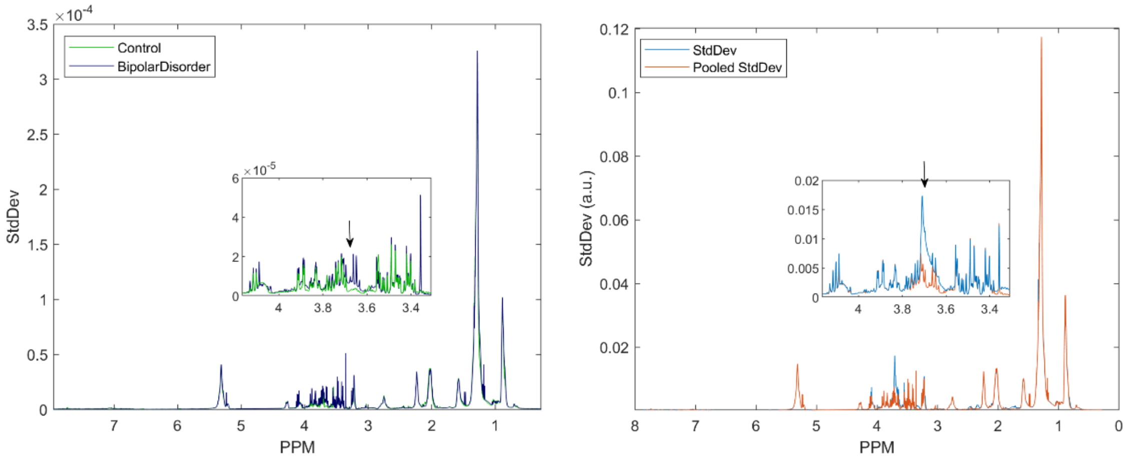

(a) **(b)**

Figure S1. (**a**) Standard deviation along all variables (chemical shifts) in the data set for the BD samples group. (**b**) Standard deviation and pooled standard deviation for all variables in the data set considering all samples from both groups (BD and controls) depending on calculation methods. Arrows in inset plots indicate the ppm range where differences between these two classes were observed in the data set.

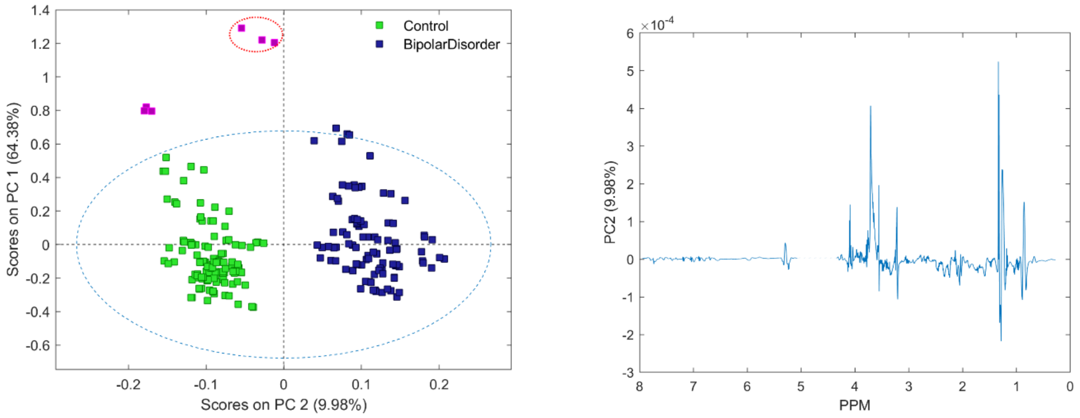

(a) **(b)**

Figure S2. (**a**) PCA score plot of the model obtained using mean centering and Pareto scaling. Samples rounded by dotted red ellipses from the BD patients group were also identified on the influence plot as potential outliers. A Hoteling T2 Confidence limit of 95% was also presented in the plot with the blue dashed line. (**b**) Loading plot of PC 2 component using mean centering and Pareto scaling.

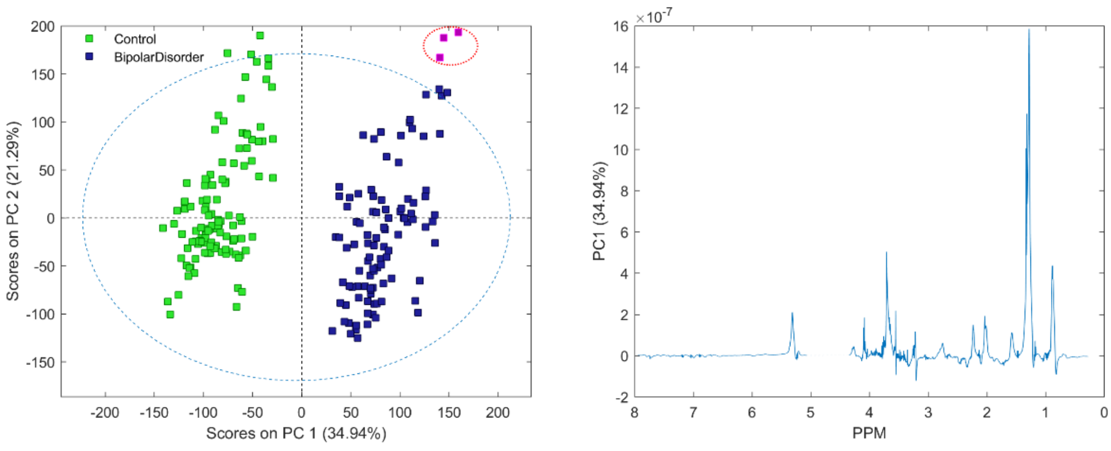

(a) **(b)**

Figure S3. (**a**) PCA score plot of the model obtained using class centroid centering and scaling. Samples marked in pink color rounded by dotted red ellipses from the BD group were also identified on the influence plot as potential outliers. (**b**) Loading plot of PC1 component using class centroid centering and scaling.

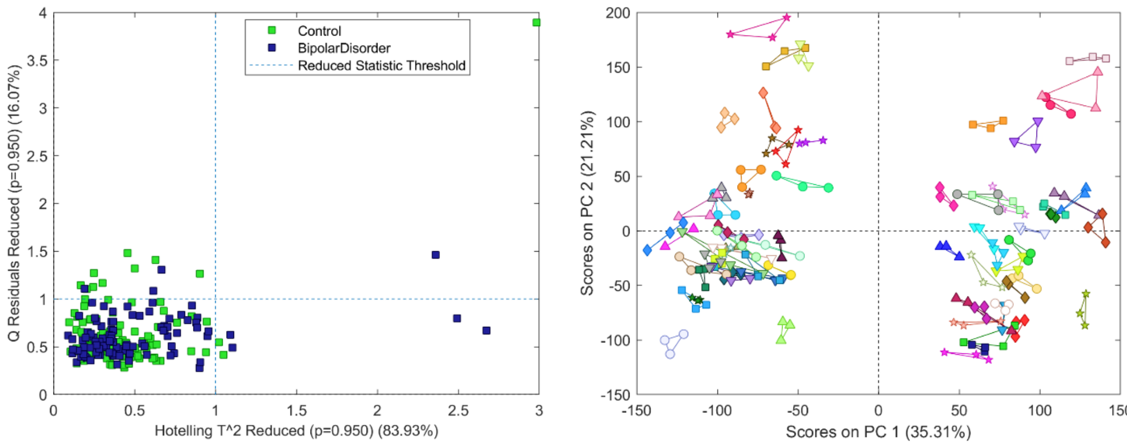

(a) **(b)**

Figure S4. (**a**) Influence plot for PCA model with class centroid centering and scaling. 3 samples from class 'BD' show large Hotelling T^2 reduced values compared to other samples and 1 from class 'Control' show higher values for both statistics. The reduced Q and T^2 are normalized statistics, divided by the confidence limit calculated from each model's particular data and parameters. (**b**) PCA score plot of the model obtained using class centroid centering and scaling, after removal of outlier samples. Scores of samples belonging to the different patients are grouped together and denoted with corresponding symbols and colors connected by lines among samples of each patient.

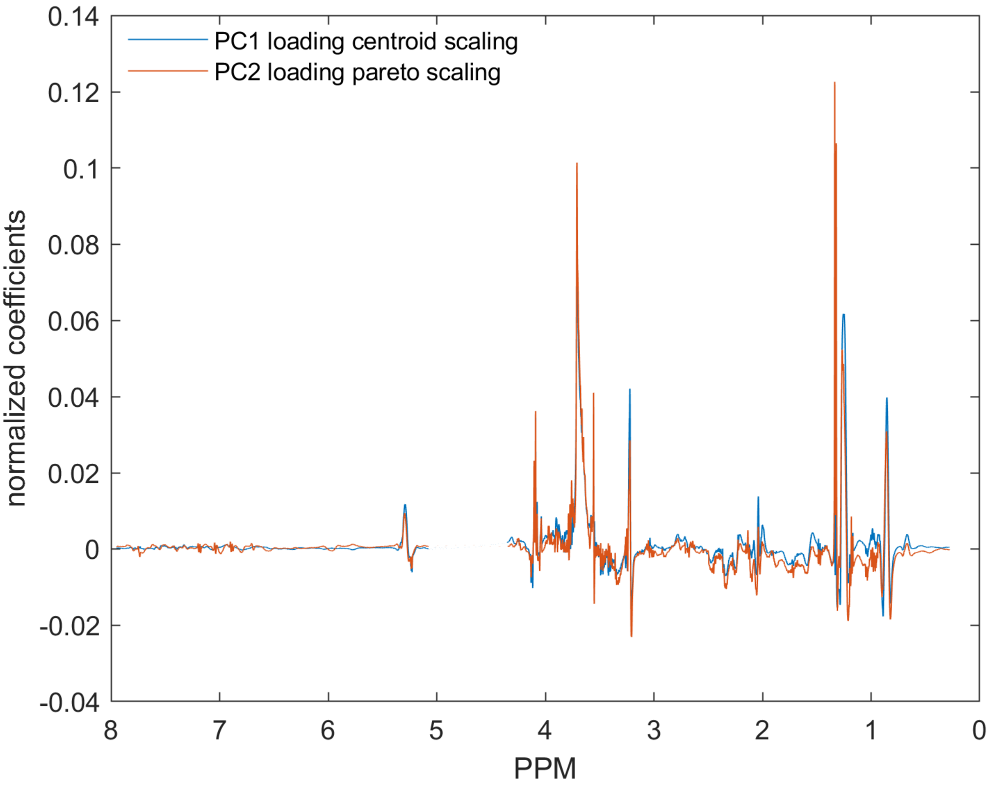

Figure S5. Loading plot of PC1 component from model preprocessed by class centroid and centering and PC2 component from model preprocessed by mean centering and pareto scaling varimax rotation of initial PCA model loadings. Resutling loadings are back transformed (multiplying all values by their respective standard deviation)
